# Pilot Study on the Effect of a Single Topical Application of Trichloroacetic Acid 85% on Normal Cervical Tissue

**DOI:** 10.3389/fphar.2022.880333

**Published:** 2022-05-20

**Authors:** Laila Nuranna, Dolly N. Lubis, Wawaimuli Arozal, Sigit Purbadi, Agian Jeffilano Barinda, Gatot Purwoto, Primariadewi Rustamadji, Andi D. Putra, Tofan W. Utami, Aria Kekalih

**Affiliations:** ^1^ Obstetrics Gynecology Department, Faculty of Medicine, University of Indonesia, Cipto Mangunkusumo National General Hospital, Oncology Gynecology Division, Jakarta, Indonesia; ^2^ Department of Pharmacology and Therapeutics, Faculty of Medicine, University of Indonesia, Jakarta, Indonesia; ^3^ Department of Pathology and Anatomy, Faculty of Medicine, University of Indonesia, Cipto Mangunkusumo National General Hospital, Jakarta, Indonesia; ^4^ Community Medicine Department, Faculty of Medicine, University of Indonesia, Jakarta, Indonesia

**Keywords:** trichloroacetic acid, cervical intraepithelial neoplasia (CIN), topical application, tissue destruction, treatment

## Abstract

**Background:** Early detection and treatment of cervical intraepithelial neoplasia (CIN) through a “see and treat” approach is a pillar of cervical cancer prevention programs in developing countries such as Indonesia. One of the major challenges faced is the limited N_2_O or CO_2_ gas supply for cryotherapy. Thus, an alternative therapeutic method such as trichloroacetic acid (TCA) topical application is needed as an alternative solution. The effectiveness of this therapy will depend on its destructive effect on eliminating the whole lesion in CIN.

**Objective:** To estimate the extent of damage in the normal cervical tissue after a single topical application of 85% TCA solution.

**Design and Methods:** This research was an intervention study carried out by applying ±5 ml of 85% TCA solution into the cervix of 40 patients scheduled for total hysterectomy for indications other than cervical pathology 24 h before surgery. The extent of tissue destruction was determined microscopically using histopathological specimens. The study protocol is registered at www.clinicaltrial.gov (ID NCT04911075).

**Results:** In the final analysis, 39 subjects were included. The necrotic area was detected at the superficial layer, accompanied by the full epithelial erosion thickness. In addition, there were also fibrotic areas resembling burned tissue in the stroma. The mean depth of destruction was 1.16 ± 0.01 mm in the anterior lip and 1.01 ± 0.06 mm in the posterior lip. There was no significant depth difference between the anterior and posterior lips (*p* ≥0.05). Moreover, the 85% TCA topical application was tolerable, as represented by the fact that the vast majority (82.1%) of participants experienced pain with a visual analog scale score of <4.

**Conclusion:** Single dose of TCA 85% in topical solution was able to destroy the normal cervical tissue with a deeper mean depth than the mean depth of CIN III in squamous epithelium.

## Introduction

Cervical cancer is becoming the fourth most common cancer, with a high mortality rate for women globally (Bray et al., 2018). In developing countries, most of the cases are detected in the late stage of the disease (Anggraeni et al., 2006-2010; Ministry of Health, 2017; WHO. Cancer, 2018) even though the natural history of cancer development would take approximately 20 years (Woodman et al., 2007; Insinga et al., 2009; Chan et al., 2019). Thus, early detection and treatment of precancerous lesions will become crucial pillars for cervical cancer prevention (WHO, 2013; World Health Organization, 2013; Adhanom Ghebreyesus, 2019). Cervical intraepithelial neoplasia (CIN) is a cervical precancerous lesion that can histologically be found in one of three stages of development (I, II, or III). The higher the degree of CIN, the deeper the precancerous lesions will be found in the epithelial lining of the cervix (Campion and Canfell, 2015). Walker et al. (2003) had identified the mean depth of CIN III lesions, which was 0.32 ± 0.70 mm. Therefore, the effectiveness of the treatment of a precancerous lesion will be determined by its ability to create tissue damage beyond the depth of the CIN III lesion (Boonstra et al., 1990). Cryotherapy using the double freeze technique is effectively reaching the desired therapeutic effect for CIN lesions (Mariategui et al., 2008; Adepiti et al., 2016). Nevertheless, limited gas supply becomes a problem for the broader application of this method (Paul et al., 2013; Direktorat Pencegahan dan Pengendalian Penyakit Tidak Menular, 2018; Budiman et al., 2019). Hence, an alternative strategy is required to substitute the limited use of cryotherapy in developing countries (WHO, 2012; WHO, 2019).

Trichloroacetic acid (TCA) is an acetic acid analog that is characterized by a transparent red crystalline solid and soluble in water with excellent solubility and stability (PubChem. Trichloroacetic acid, 2020). TCA solution can be stored in amber glass bottles and be kept in the refrigerator for up to 23 weeks (Spinowitz and Rumsfield, 1989). Currently, the potential toxicity of topical TCA in humans is considered at low risk (Brody et al., 2000; Wiley et al., 2002; Yanofsky et al., 2012; Ayres, 1962; Resnik, 1984; Patel et al., 2017). Nonetheless, a minor burning sensation and ulceration were observed in the patient treated with 85% TCA solution (Taner et al., 2007). The principle of TCA therapy is the destruction of the epidermis and dermis due to necrosis accompanied by re-epithelialization and stimulation of new collagen formation. The intensity of the necrosis depends on the concentration used. The higher the concentration, the more rapid and profound the tissue destruction (Rakic et al., 2000; Yonei et al., 2007; Kimura et al., 2011; Brodland et al., 1989; Brodland et al., 1989; Rakic et al., 2000; Yonei et al., 2007; Kimura et al., 2011). Nevertheless, the evidence for the efficacy of 85% TCA in cases of cervical precancerous lesions has not been widely studied (Geisler et al., 2016; Suwartono, 2019). Furthermore, no studies have been found to analyze the depth of destruction levels of 85% TCA solution on normal cervical tissue.

## Materials and Methods

### Design, Location, and Time

The experimental research design had received approval from the Research Ethics Committee of the FKUI/RSCM Jakarta, Indonesia. The implementation time was from January to October 2021.

### Population and Sample

The target population of the study was all patients who would undergo a total elective hysterectomy at the Obstetrics and Gynecology Department of the Faculty of Medicine/RSCM, which was not associated with a diagnosis of cervical pathology, whether benign, precancerous, or invasive carcinoma. The sample size is set at 40 people, which is obtained from the following formula:

$$N={\left\{\frac{\left(\mathrm{Z\alpha}\right)\mathrm{x SD}}{d}\right\}}^{2}$$

Notes:

1) N: minimum sample size for each treatment group
2) Z: coefficient for confidence level *α* = 5% (1.96)
3) SD: standard deviation of examination results 0.64 (Adepiti et al. (2016), projections from single freeze cryotherapy
4) d: absolute error 5% of the average depth of 4.2 mm (0.21 mm)

**FIGURE 1:**
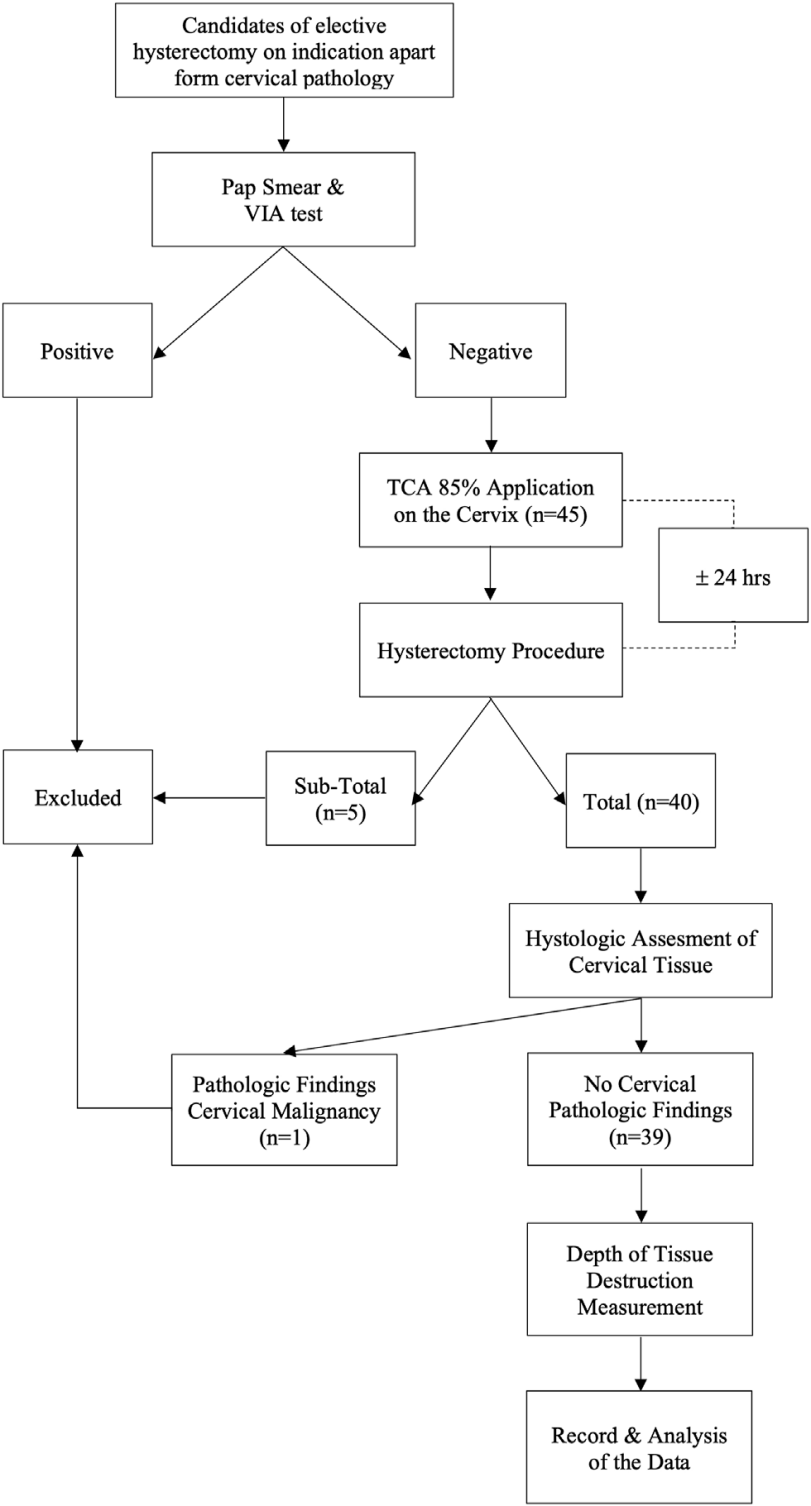
Flow chart of the study.

Inclusion criteria were normal cervix determined from clinical, radiological examinations, and negative visual inspection with acetic acid (VIA) test results. We consider the use of the VIA test to determine a normal cervix to be equivalent to the HPV test and pap-smear. According to a previous study, in subjects with a negative VIA test result, the chance of HPV infection was only 3.21 percent (Utami, 2016). VIA test has also been shown to be as effective as cytological examination for detecting cervical pre-cancerous lesions, with a sensitivity level of 90% and specificity of 92.2 percent. (Sankaranarayanan et al., 1998). Meanwhile, the participants who underwent a subtotal hysterectomy procedure or in whom cervical pathology was detected at the histological evaluation were excluded from the study. The flow chart of this study is depicted in Figure 1.

### Intervention

**FIGURE 2:**
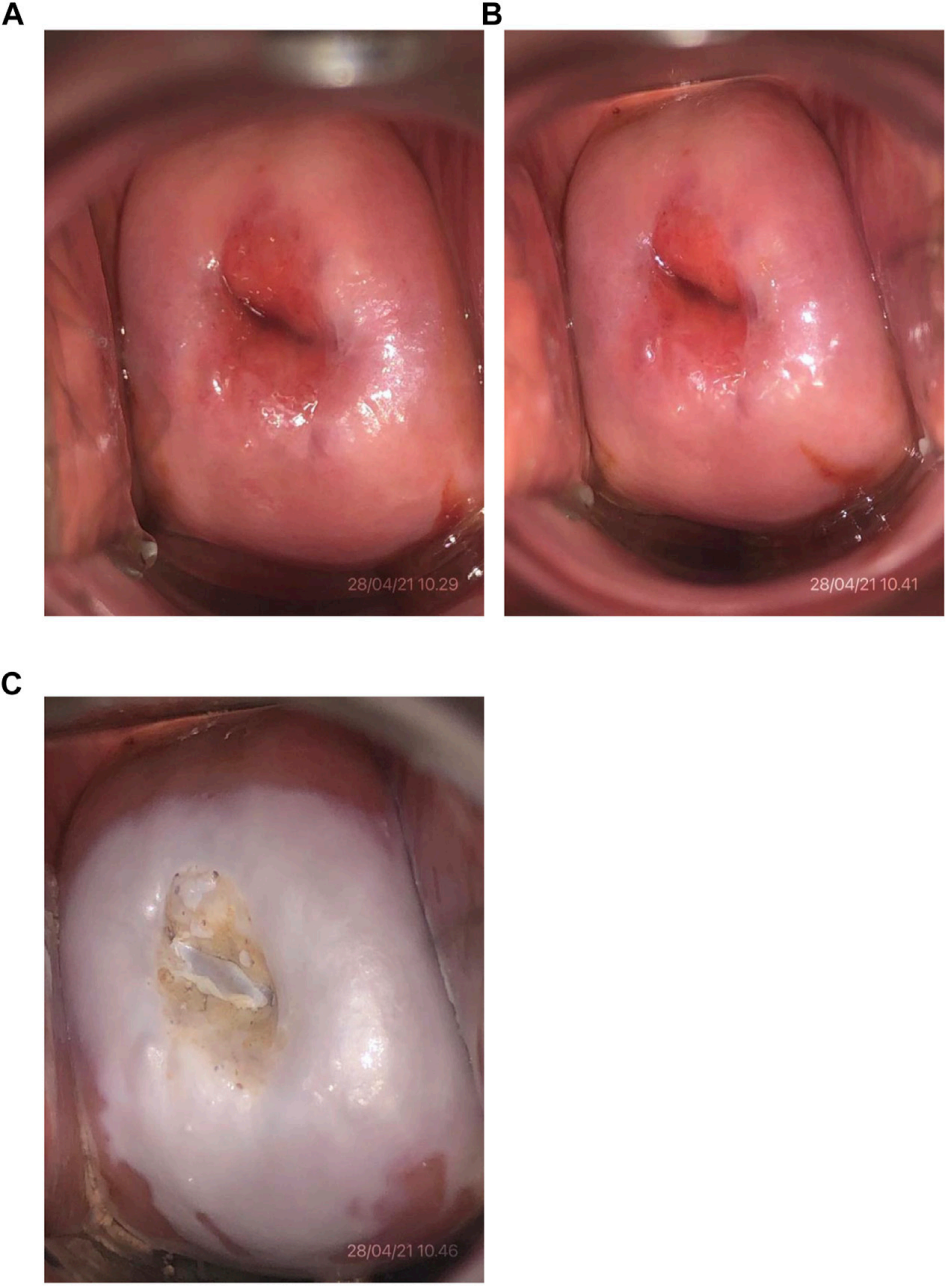
Cervical photo after 85% TCA application. **(A)** Initial cervical condition of one of the subjects (No. 4). **(B)** Condition after visual inspection with acetic acid test with a negative result. **(C)** Condition after application of 85% TCA solution.

The 85% TCA solution in this study was made according to the standard guideline from the Pharmacology Laboratory of the Faculty of Medicine/RSCM by using the weight-in-volume method (Harmon et al, 2006). Tools and materials utilized for making the solutions were previously sterilized using an autoclave. The 85% TCA solution was prepared by dissolving 85 g of pure crystal TCA (EMSURE^®^ Merc^®^) in 100 ml of pure distilled water and stirring until homogenous. The solutions were freshly prepared before the subjects underwent the procedure. Solutions that were not used within seven days were discarded and replaced with new solutions. Twenty-four hours before the surgery, ± 5 ml of 85% TCA solution was applied to the cervix using a cotton swab by one investigator (DNL). The smear was made thinly into the ectocervical area, and the transformation zone appeared after a light pressing for 1–3 min until a white appearance occurred, indicating the precipitate of protein denaturation. For the endocervical canal, the tip of the stalk of a small cotton swab was dipped in the solution and inserted through the external uterine ostium (Figure 2).

### Data Collection

Hysterectomy was performed 24 h after TCA application. A pathologist excised the cervix from the uterus and cut at the 12 and 6 o’clock positions to separate the left and right lips. Both pieces of tissue were fixed in formalin for 24 h. Multiple sections consisting of anterior and posterior division were obtained from each lip. The microscopic evaluation was focused on the measurement of the depth of necrosis with 40× magnification. The slides were then analyzed using *ImageJ®* (www.imagej.nih.gov) image processing software. The criterion to determine the deepest level of necrosis was based on the destruction of the glandular epithelium in the gland crypt in the stroma or the destruction of the endothelium of the stromal blood vessels (Mariategui et al., 2008; Adepiti et al., 2016). The final result of the measurement was corrected by adding 20% of the value to compensate for shrinkage from preanalytic tissue processing (Boonstra et al., 1990; Walker et al., 2003). This study protocol is registered at www.clinicaltrials.gov ID NCT04911075.

### Data Analysis

The statistical analysis was performed using SPSS version 26. The data were presented as mean with a standard error of the margin (SEM). The difference between groups was analyzed using a two-tailed t-test. Correlation among the variables was assessed *via* the point biserial correlation test. Statistical significance was considered with *p* <0.05.

## Results

### Subject Characteristics

Thirty-nine subjects were included in the final analysis. One subject was excluded because a cancerous lesion was observed in the cervical stroma. Table 1 depicts the baseline characteristics. The mean age was 51.3 ± 1.2 years. Overall, 43.6% of the subjects had a body mass index (BMI) above the normal range. Over half (51.3%) of the subjects were still menstruating, and the majority (79.5%) had a history of childbirth. Macroscopically, the average cervical length was 37.2 ± 1.1 mm with a diameter of 27.1 ± 0.8 mm. Only 17.9% of the subjects complained of pain with a visual analog scale (VAS) score of ≥4 at the time of application. The time interval between TCA exposure and tissue fixation was 26.3 ± 1.4 h.

**TABLE 1:** Subject characteristics.

Variable		Value (*n* = 39)
Age (years)	–	51.26 ± 1.20^a^
–	•≤50	22 (56.4%)
–	•>50	17 (43.6%)
BMI (kg/m^2^)	–	25.53 ± 5.25^a^
–	•<25	22 (56.4%)
–	•≥25	17 (43.6%)
Menopausal status	•Not yet	20 (51.3%)
–	•Yes	19 (48.7%)
Parity	•Nulliparous	8 (20.5%)
–	•Parous	31 (79.5%)
VAS score	•<4	32 (82.1%)
–	•≥4	7 (17.9%)
Interval TCA-fixation (h)	–	26.25 ± 1.37^a^
Cervical length (mm)	–	37.21 ± 1.11^a^
Cervical diameter (mm)	–	27.08 ± 0.78^a^

a Mean value ± SEM.

### Microscopic Appearance of Tissue Destruction

**FIGURE 3:**
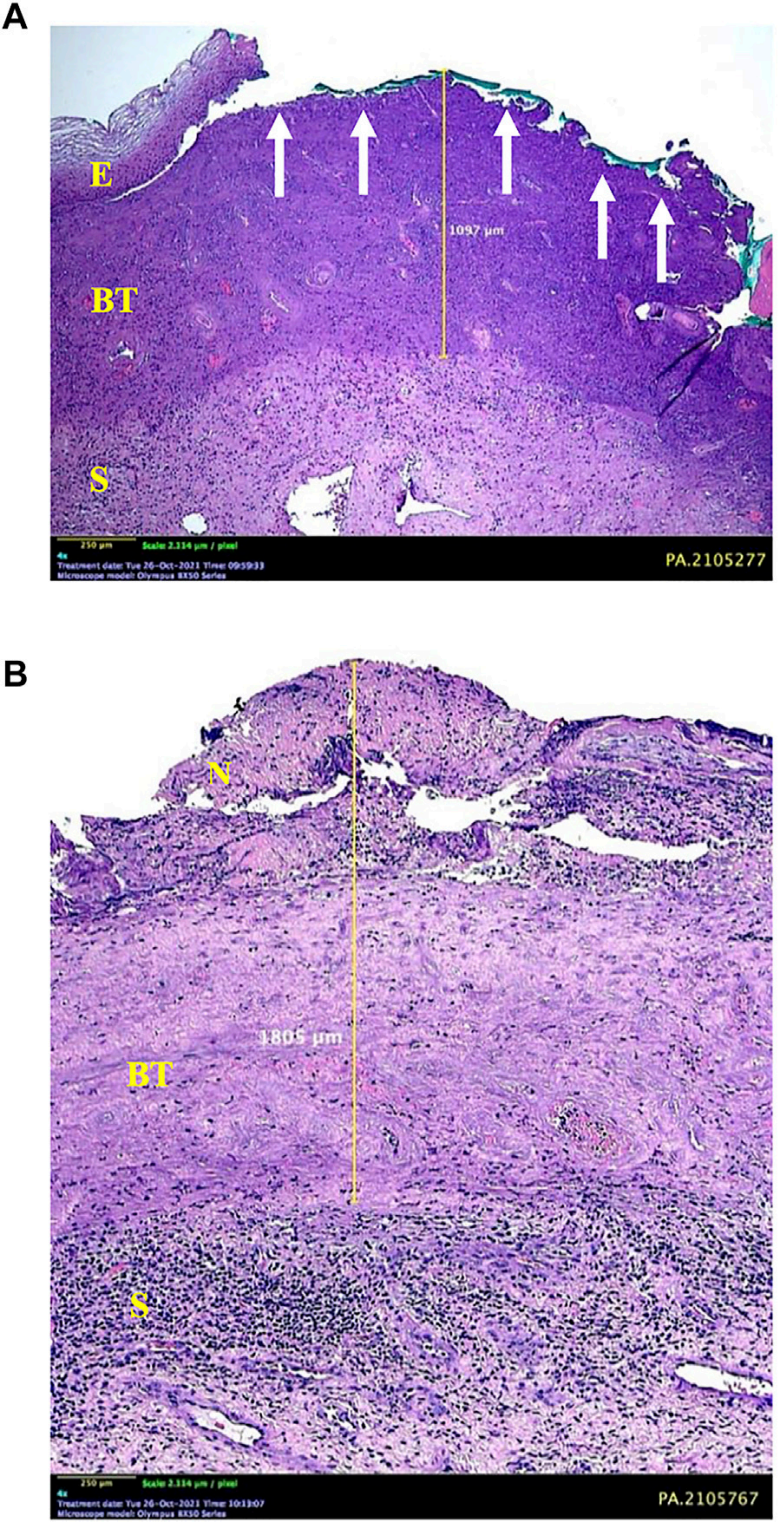
Microscopic appearance of cervical tissue destruction after 85% TCA application. **(A)** Some areas show erosion of the entire thickness of the epithelium (E) (arrows) with underlying burn tissue to a depth of 1.097 mm. **(B)** Superficial layer of necrosis (N), which continues with a burn-tissue–like layer into the stroma (S). A boundary between the layer of burn tissue and normal tissue accompanied by a massive infiltration of inflammatory cells is observed at the bottom. This is the limit for measuring depth, which is 1.805 mm.

Extensive areas of necrotizing lesions in the superficial layer were detected, accompanied by erosion of the full thickness of the epithelium in several places. Some histological samples found a fibrotic area of stroma resembling a burn wound under the superficial necrosis layer. Moreover, the burn-like tissue was bordered by the normal cervical stroma massively infiltrated with inflammatory cells (Figure 3).

### Tissue Destruction Depth Analysis

**FIGURE 4:**
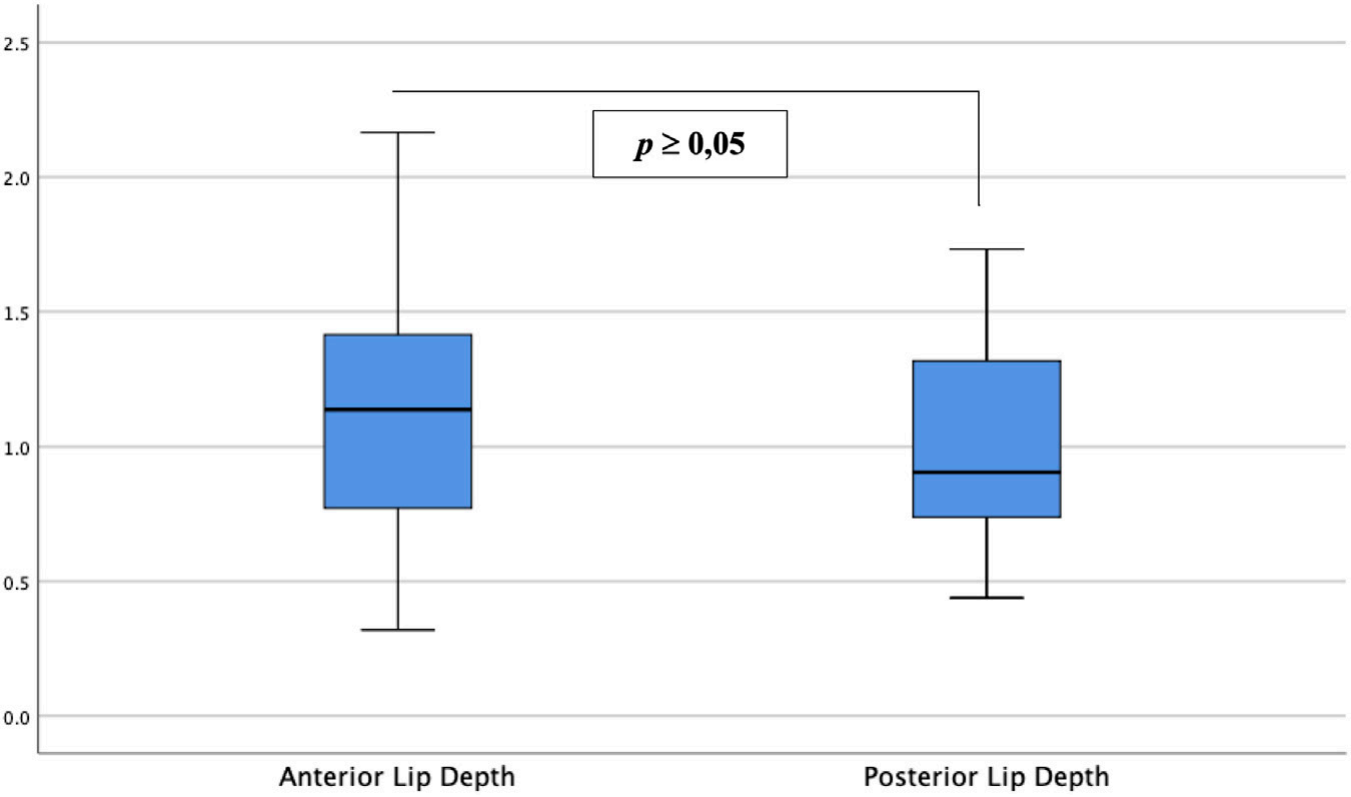
Depth of anterior and posterior cervical lip tissue destruction. Between the two groups, there was no significant difference in depth of tissue destruction between anterior and posterior cervical lips (*p* ≥0.05).

Table 2 and Figure 4 show the analyses of the depth of tissue destruction for all subjects and their description of data, respectively. There was no significant difference between the average depth of anterior and posterior lip tissue destruction (1.16 ± 0.01 vs. 1.01 ± 0.06 mm; *p* ≥0.05). The subgroup analysis also found a comparable average in age group, menopausal status, and parity status (*p* ≥0.05). Meanwhile, the difference was significant only in the anterior lip (1.30 ± 0.08 vs. 0.97 ± 0.08 mm; *p* <0.05), adjusted by the BMI (Table 3). Correlative analyses among the factors showed that age, menopausal status, length, and diameter of the cervix were not correlated to the depth of necrosis, both in the anterior and posterior lips (*p* ≥0.05). Conversely, BMI and parity status only showed a moderate negative correlation to the depth of the anterior cervical lip (*p* <0.05). (Table 4).

**TABLE 2:** Depth of anterior and posterior cervical lip tissue destruction.

Statistics	Anterior lip (*n* = 39)	Posterior lip (*n* = 39)	*p*-Value
Mean ± SEM (mm)	1.16 ± 0.01	1.01 ± 0.06	≥0.05^a^
Minimal depth (mm)	0.32	0.44	
Maximal depth (mm)	2.17	1.73	
Range (mm)	1.85	1.29	

a Independent t-test (*α* = 0.05).

**TABLE 3:** Differences in the mean depth of tissue destruction based on subject characteristics.

Variable		Anterior lip (*n* = 39)		Posterior lip (*n* = 39)	
		Mean ± SEM (mm)	*p*-Value	Mean ± SEM (mm)	*p*-Value
Age (years)	•≤50	1.09 ± 0.07	≥0.05^b^	1.00 ± 0.06	≥0.05^a^
	•>50	1.24 ± 0.09		1.02 ± 0.05	
BMI (kg/m^2^)	•<25	1.30 ± 0.08	**<**0.05^a^	1.09 ± 0.06	≥0.05^a^
	•≥25	0.97 ± 0.08		0.90 ± 0.06	
Menopausal status	•Not yet	1.12 ± 0.07	≥0.05^a^	0.97 ± 0.05	≥0.05^a^
	•Yes	1.20 ± 0.08		1.05 ± 0.06	
Parity	•Nulliparous	1.47 ± 0.11	≥0.05^b^	1.02 ± 0.06	≥0.05^a^
	•Parous	1.08 ± 0.07		1.01 ± 0.06	

a Independent t-test (*α* = 0.05).

b Independent Mann–Whitney U test (*α* = 0.05).

**TABLE 4:** Analysis of factors affecting the depth of tissue destruction.

Variable	Anterior lip		Posterior lip	
	Correlation coefficient	*p*-Value	Correlation coefficient	*p*-Value
Age (years)	−0.06	≥0.05^a^	0.85	≥0.05^a^
BMI (kg/m^2^)	−0.37	<0.05^b^	−0.28	≥0.05^b^
Menopausal status	0.87	≥0.05^c^	0.11	≥0.05^c^
Parity	−0.33	<0.05^c^	−0.01	≥0.05^c^
Cervical length	−0.14	≥0.05^b^	−0.11	≥0.05^b^
Cervical diameter	−0.16	≥0.05^a^	0.04	≥0.05^a^

a Pearson correlation test (*α* = 0.05).

b Spearman correlation test (*α* = 0.05).

c Point biserial correlation test (*α* = 0.05).

## Discussion

To the best of our knowledge, this is the first study that analyzed the depth of necrosis in normal cervical tissue after a single topical application of 85% TCA solution. The pathophysiology of tissue destruction as the effect of TCA on epithelial tissue has been described in previous studies. This solution can cause protein denaturation, leading to cell necrosis accompanying an inflammatory reaction. A study by Rakic et al. (2000) showed that TCA was cytotoxic for keratinocytes and depressed protein–collagen synthesis and the expression of matrix metalloproteinases (MMPs) production of human dermal fibroblasts. In human skin, according to Yonei et al. (2007), platelet-derived growth factors subunit B (PDGF-B) mRNA expression became significantly upregulated after TCA application and then immediately downregulated. Immunoreactive PDGF-B in the cytoplasm of keratinocytes became detectable throughout the epidermis after TCA application, reached a maximum after the peak of mRNA expression, and then declined significantly over 24 h when the epidermis became completely necrotic. Kimura et al. (2011) stated that after TCA treatment, transient upregulation of proopiomelanocortin (POMC) and melanocortin receptor 1 (MC1R) mRNA expressions were observed in human skin. These results suggest that TCA activates the skin stress response system by inducing POMC and MC1R productions in keratinocytes. In this study, histologically, the necrotic area was detected in the superficial layer, accompanied by the full thickness of the epithelium erosion. In addition, there were areas of fibrotic stroma resembling burned tissue surrounding the normal stromal tissue with massive inflammatory cells. This appearance will undergo necrosis later (Sankaranarayanan et al., 1998; Harmon et al., 2006; Leopold et al., 2012).

The detailed measurement of the extent of necrotic tissue was analyzed. The depth of necrosis after application of 85% TCA was (1.16 ± 0.01 mm) in the anterior lip of the cervix and (1.01 ± 0.06 mm) in the posterior, respectively. Yonei et al. (2007) described that the cytotoxic effect of TCA was closely associated with the concentration of the solution. Another study further confirmed these data that showed the average depth destruction level of 80% TCA solution was 0.98 ± 0.17 mm (Brodland et al., 1989). In this study, the higher TCA concentration (85%) may achieve deeper necrosis in the normal tissue. Compared with the previous study (Walker et al., 2003), the deeper destruction induced by 85% TCA may reach beyond the average depth of CIN I (0.30 ± 0.93 mm), CIN II (0.36 ± 0.91), and even CIN III (0.32 ± 0.70 mm).

The comparable mean depth between anterior and posterior lip necrosis was also important. There was a discrepancy in the necrotic depth among anterior and posterior lips in the previous study. This fact could represent the noninferiority of TCA therapy compared with cryotherapy (Mariategui et al., 2008; Adepiti et al., 2016). In fact, several studies have also shown that the topographic positions in the cervix may influence the CIN lesions (Pastar et al., 2014). Of note, most severe lesions were found at the 8 and 7 o’clock positions, either in low- or high-grade lesions. In a similar manner, Zhao et al. (2015) identified that quadrants 2 and 3, specifically in the 4 and 7 o’clock positions, were susceptible to CIN on the cervix. CIN will be found more frequently on the posterior lip than on the anterior lip of the cervix. Thus, a therapeutic modality that can provide an equal depth effect between the anterior and posterior lips will be beneficial.

The potential of using an 85% TCA solution for therapy is also supported by the data that most of the subjects (82.1%) only experienced pain with a VAS score of less than four. Similar to the previous study, the main complaint of topical TCA application was mild pain or burning sensation. (Wiley et al., 2002; Yanofsky et al., 2012). However, no significant side effects were found in this study.

Several factors were thought to affect the depth of penetration level of TCA into cervical tissue, which was analyzed as an additional outcome in this study. The clinical characteristics of the subjects, particularly age or menopausal status, may influence the destruction level, although the results of previous studies were controversial. According to the study conducted by Mariategui et al. (2008), the cervical necrotic depth in the younger subjects was shallower due to the sufficient vascularity in the cervical area, thus making them resistant to hypoxic conditions. Conversely, the necrotic area may become shallower in the elderly or postmenopausal subjects due to increased tight junctional resistance. This factor becomes a major determinant for epithelial permeability that maintains the integrity and organization of cells through the action mechanism of various proteins, such as claudins (Blaskewicz et al., 2011; He et al., 2012; Zhao et al., 2015). Nevertheless, in this study, no association was found between age or menopausal status with the mean depth of tissue destruction or cervical physical dimensions, including length and diameter.

BMI and parity status may affect the extent of the destruction. Previous studies had shown that BMI and parity status might influence ovarian hormone levels, especially estrogen. Overweight and nulliparity are risk factors for higher estrogen levels (Barrett et al., 2014, Espetvedt Finstad et al., 2009, Lee et al., 2005). Gorodeski et al. (2000) stated that changing estrogen levels partly affected the transcervical paracellular permeability. This phenomenon, in turn, may have an influence on TCA’s penetration depth in cervical tissue. In this study, BMI and parity status were negatively correlated with the depth of necrosis. Nonetheless, the strength of the relationship that occurred is not so convinced and only occurred on the anterior lip. Based on these results, we conclude that the subject’s clinical and physical characteristics of their cervix did not significantly affect the depth of TCA’s tissue destruction in this study.

The strength of this study lies in the method of examining the depth of cervical tissue destruction, which is carried out microscopically through tissue histologic analysis. The data on the depth of tissue destruction from this study can be used as the evidence base literature for the application of 85% TCA in cervical precancer cases found through a “see and treat” approach as an alternative to cryotherapy. However, the limitation of this study was the insufficiency of samples number for performing subgroup analysis parametrically. A study with a larger number of samples is needed for achieving a better understanding of TCA’s depth penetration correlating factors.

## Conclusion

The preliminary data on the destruction levels of a single topical application of 85% TCA in the normal cervical tissue can be obtained through this study, which is 1.16 ± 0.01 mm in the anterior lip and 1.01 ± 0.06 mm in the posterior, respectively. These results are deeper than the reference of CIN III mean depth in the cervical squamous epithelium. Based on the results of this pilot study, it is interesting to design a future study to determine the depth of tissue destruction through the application of a single dose of 85% TCA in cases of cervical pre-cancerous lesions (CIN).

## Data Availability

The original contributions presented in the study are included in the article/Supplementary Material, further inquiries can be directed to the corresponding author.
